# Nonlinear response speedup in bimodal visual-olfactory object identification

**DOI:** 10.3389/fpsyg.2015.01477

**Published:** 2015-09-30

**Authors:** Richard Höchenberger, Niko A. Busch, Kathrin Ohla

**Affiliations:** ^1^Psychophysiology of Food Perception, German Institute of Human Nutrition Potsdam-Rehbrücke (DIfE)Nuthetal, Germany; ^2^Institute of Medical Psychology, Charité - Universitätsmedizin BerlinBerlin, Germany; ^3^Berlin School of Mind and Brain, Humboldt UniversityBerlin, Germany

**Keywords:** multisensory integration, olfaction, visual-olfactory, race model, response time

## Abstract

Multisensory processes are vital in the perception of our environment. In the evaluation of foodstuff, redundant sensory inputs not only assist the identification of edible and nutritious substances, but also help avoiding the ingestion of possibly hazardous substances. While it is known that the non-chemical senses interact already at early processing levels, it remains unclear whether the visual and olfactory senses exhibit comparable interaction effects. To address this question, we tested whether the perception of congruent bimodal visual-olfactory objects is facilitated compared to unimodal stimulation. We measured response times (RT) and accuracy during speeded object identification. The onset of the visual and olfactory constituents in bimodal trials was physically aligned in the first and perceptually aligned in the second experiment. We tested whether the data favored coactivation or parallel processing consistent with race models. A redundant-signals effect was observed for perceptually aligned redundant stimuli only, i.e., bimodal stimuli were identified faster than either of the unimodal components. Analysis of the RT distributions and accuracy data revealed that these observations could be explained by a race model. More specifically, visual and olfactory channels appeared to be operating in a parallel, positively dependent manner. While these results suggest the absence of early sensory interactions, future studies are needed to substantiate this interpretation.

## 1. Introduction

Olfactory and visual sensory information are continuously flooding the brain and are, therefore, often experienced with a marked temporal overlap or even simultaneously. Both the smell and visual appearance serve a vital function in the localization of food, the assessment of edibility, as well as the identification of potential environmental hazards, thereby allowing for fast and appropriate behavior not only limited to food-choice. The integration of redundant sensory information by the neural system has been proven beneficial for perception and subsequent behavior: it speeds up processing and improves accuracy. However, it is unclear whether this holds true for the combination of olfaction and vision.

Recent studies have shown that odors modulate visual perception and performance, particularly by directing attention to and influencing the saliency of a congruent visual object, e.g., during attentional blink (Robinson et al., 2013), binocular rivalry (Zhou et al., 2010, 2012), spatial attention and visual search (Chen et al., 2013), and eye movements (Seo et al., 2010). These effects occur even when odors are task-irrelevant and suggest spontaneous binding between visual and olfactory inputs (Zhou et al., 2012). In contrast, odor perception is not only influenced by vision (and the other senses), but odor identification also critically depends on additional information because odors in isolation are notoriously ambiguous (Cain, 1979). Observations that humans have difficulties identifying and discriminating odors in the absence of additional information (Davis, 1981) and that color cues (Zellner et al., 1991), verbal labels (Herz and von Clef, 2001) and images (Gottfried and Dolan, 2003) assist odor perception corroborate this notion. Most previous studies investigated modulatory effects of visual cues on olfaction and their interaction at cognitive levels, when semantic representations were available. It remains unknown whether sensory information from the olfactory and visual modalities is in fact pooled at early perceptual stages, that is, integrated.

### 1.1. How can we investigate whether multisensory integration is taking place?

Multisensory integration has been mostly studied between the non-chemosensory modalities vision, hearing, and somatosensensation; these senses have been shown to interact already at the level of the superior colliculi (Stein and Meredith, 1990). Classically, super-additive responses, i.e., more than the sum of the parts, are considered an indication of multisensory integration. Key aspects governing multisensory integration are the so-called principles of spatial and temporal proximity: stimuli presented at the same location and at the same time, respectively, most likely belong to the same object and are therefore more likely to be bound together to a unitary percept (Stein and Meredith, 1993). Additionally, Meredith and Stein (1986) found that cells in the superior colliculus produced the strongest response amplification for the weakest stimuli, a principal phenomenon called *inverse effectiveness*. While these observations could be replicated on the behavioral level in numerous studies, it has been suggested that these findings might largely be statistical artifacts (Holmes, 2007). While imaging studies have mostly focused on superadditive effects when trying to identify functional correlates of multisensory integration, it is unclear whether the results from single-neuron recordings can be readily transferred to the cortical level (Laurienti et al., 2005) and behavior.

### 1.2. Response facilitation can serve as a possible measure of multisensory integration

Stimulus detection, on average, is faster and more accurate in situations where the target is presented redundantly, i.e., on several sensory channels. In the multisensory literature, this facilitation is commonly called *redundant-targets effect* (RTE) or *redundant-signals effect* (RSE); both terms are largely used synonymously. In the remainder of this paper, we will refer to these effects of multisensory processing exclusively as redundant-signals effects. The response speedup is commonly explained by assuming that an internal decision criterion is reached faster when multiple targets are presented simultaneously, compared to the single-target situation. Similarly, redundant information reduces stimulus ambiguity, hence allowing for a higher accuracy of responses.

However, RSEs can result from *statistical facilitation* merely due to probability summation alone. A popular probability summation model was introduced by Raab (1962) with the idea of a *race* between parallel single-target detection processes during a multiple-target situation. The process finishing first “wins the race,” elicits a response, and, therefore, determines the behavioral response time. These so-called race models operate according to a *separate-activation model* with a *first-terminating stopping rule* (see e.g., Colonius and Vorberg, 1994). They implicitly assume unlimited-capacity processing (Colonius, 1990), meaning that the speed of one detection process is not influenced by other, simultaneous, detection processes. For example, detection of a unimodal target should happen at the same speed as detection of the same target in a multimodal situation. Therefore, if RT distributions of the single-target detection processes overlap, the observed RTs in redundant-target trials will, on average, be faster than the unimodal RTs. “Slow” responses of one single-target detection process can be replaced by “faster” responses of another, simultaneous detection process. The observed RT speedup would thus be a statistical artifact only. In sum, an RSE that can be fully accounted for by a race model does not provide strong evidence for multisensory integration.

Nevertheless, integration can be inferred if RTs are faster than predicted by race models. Specifically, Miller (1982) derived an upper bound to the bimodal RT speedup possible in *any* race model, the so-called *race model inequality* (RMI) or *Miller bound*. It is based on the assumption of maximum negative dependence between the channel processing speeds (Colonius, 1990), that is, if the participant detects a signal on one channel at a given bimodal trial, the other channel will fail to detect the target. Violations of this criterion, i.e., faster responses than predicted by the RMI, support *coactivation models*. They demand that processing of different sensory channels be pooled prior to the decision stage and therefore refute all race models in favor of “true” multisensory integration. Satisfaction of the RMI, on the contrary, does not necessarily exclude coactive processing.

Numerous studies have investigated response facilitation to bimodal stimuli in the visual, auditory, and somatosensory modalities (see e.g., Gielen et al., 1983; Miller, 1986; Forster et al., 2002; Diederich and Colonius, 2004). Whether the combined presentation of congruent (that is, redundant) visual-olfactory information can likewise facilitate object perception remains unclear and was investigated with the present study. Specifically, we tested the hypothesis that bimodal visual-olfactory object identification is facilitated compared to identification of either of the unimodal constituents alone.

Furthermore, we examined whether facilitation is more pronounced for perceptually aligned compared to physically aligned stimuli. For this, we conducted two experiments in which the bimodal constituents were either presented physically (Experiment 1) or perceptually (Experiment 2) simultaneously. We compared the observed RTs and response accuracies to the predictions of different models of probability summation.

## 2. Results

**Figure 1:**
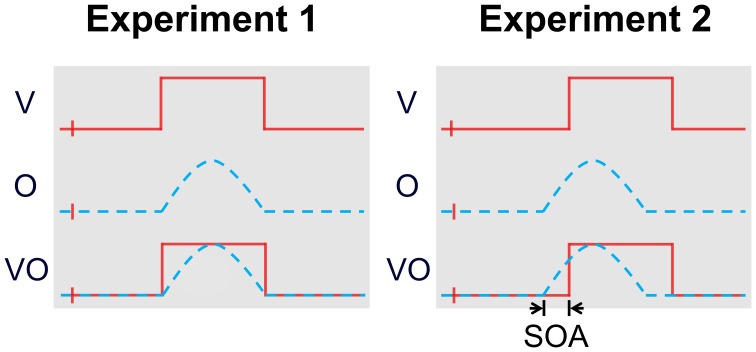
**Stimulus timing in unimodal and bimodal trials of Experiments 1 and 2 (schematic)**. The start of a trial is marked with a small vertical line and identifies the onset of the fixation cross. The visual and olfactory constituents were presented physically simultaneously in the bimodal trials in Experiment 1, and perceptually simultaneously in Experiment 2. The SOAs employed in Experiment 2 were individually estimated for every participant and object (banana and lemon, respectively) during Experiment 1. Note that the depicted delayed presentation of the visual stimulus in Experiment 2 caused the fixation cross to be displayed for a longer duration before stimulus onset in the unimodal vision-only condition in order to ensure context-invariance.

Seven participants smelled and viewed different food objects presented either alone as unimodal visual (V) or olfactory (O) stimuli or as congruent bimodal combinations (OV) and performed a speeded two-alternative forced-choice (2-AFC) object identification task. Stimulus strength was adjusted to achieve approximately 75 % accuracy. The biggest RSE for response time (RT) can be observed when the RT distributions of the unimodal constituents overlap largely (Hilgard, 1933; Hershenson, 1962; Raab, 1962; Miller, 1986; Colonius, 1990; Diederich and Colonius, 2004; Gondan, 2009). Therefore, we conducted two separate experiments, in which OV stimuli consisted of *physically* (Experiment 1; Figure 1, left) or *perceptually* (Experiment 2; Figure 1, right) aligned unimodal constituents. Perceptual alignment was achieved by introduction of a stimulus-onset asynchrony (SOA) equal to the RT differences between the unimodal stimuli.

**Figure 2:**
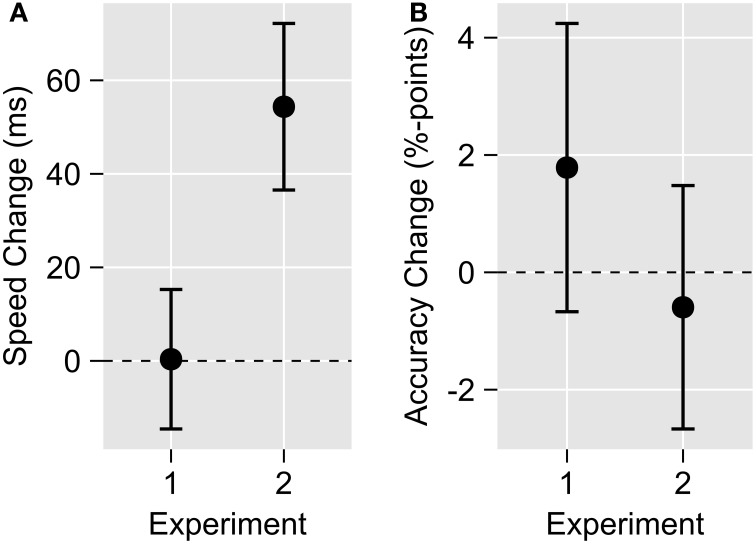
**Mean redundant-signals effects in Experiments 1 and 2**. Positive values indicate a bimodal facilitation, negative values a bimodal impairment relative to the unimodal constituents. **(A)** No redundancy gain in response speed was observed for physically simultaneous bimodal stimulation (Experiment 1), but it was clearly evident for perceptually simultaneous stimulation (Experiment 2). **(B)** For both physically simultaneous (Experiment 1) and perceptually simultaneous bimodal stimulation (Experiment 2), no significant accuracy improvement could be observed. Data were calculated individually for each participant and object, and subsequently averaged. Error bars show standard error of the mean.

For perceptually aligned OV stimuli (Experiment 2) we observed a significant RSE for RTs [RSE = 54ms, *t*_(6)_ = 3.05, *p* = 0.02], that is a speedup of median RTs to bimodal OV compared to the fastest unimodal stimuli (Figure 2A). No RSE was found for physically aligned OV stimuli during Experiment 1 [RSE = 0ms, *t*_(6)_ = 0.02, *p* = 0.98]. Accuracy showed no RSE in either Experiment [Experiment 1: RSE = 1.8%-points, *t*_(6)_ = 0.73, *p* = 0.49; Experiment 2: RSE = −0.6%-points, *t*_(6)_ = −0.29, *p* = 0.78; Figure 2B].

**Figure 3:**
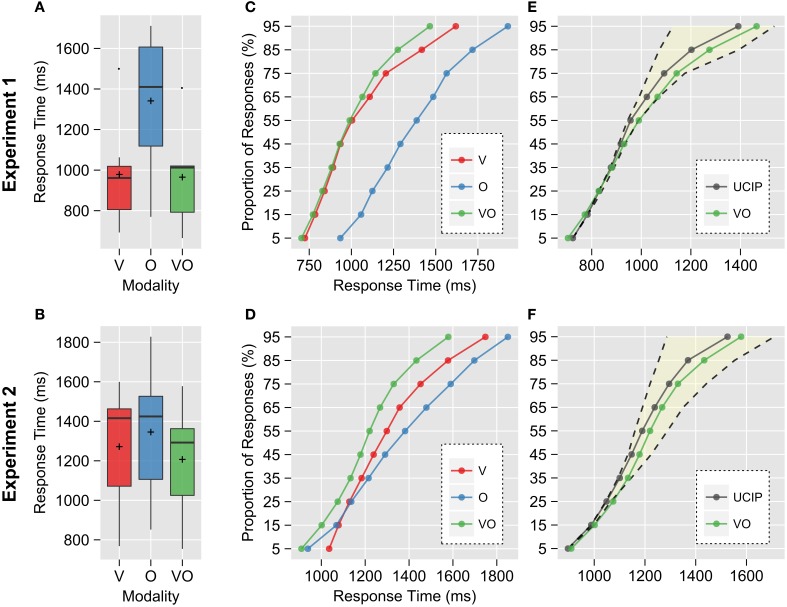
**Comparison of observed response times (RTs) in the visual (V), olfactory (O), and bimodal (VO) trials**. Visual RTs were corrected for SOA. **(A)** RT distributions for V and VO appeared to be almost identical, with the O distribution shifted to much slower RTs and very little overlap with V, indicating that responses in VO trials were mostly driven by the visual constituent. The crosses depict group means. **(B)** Individual timing adjustments by introduction of an SOA aligned the unimodal distributions, suggesting perceptual simultaneity. **(C,D)** Empirical cumulative RT distributions. Quantile values were averaged across participants. In Experiment 1, the VO distribution seemed to follow V up to the 55% quantile. Approximately at the same time, the fastest olfactory responses could be observed, i.e., the unimodal constituents were starting to perceptually overlap. Coincidentally, the VO distribution started to diverge from V, and shifted to faster responses. This bimodal speedup is not reflected in the global (mean) RSE. In Experiment 2, the VO distribution was shifted to faster responses relative to both unimodal distributions across its whole range. **(E,F)** Comparison of the unlimited capacity, independent, parallel (UCIP) model prediction with the observed bimodal data. The highlighted area depicts the possible phase space under the assumption of separate-activation models with unlimited capacity and a first-terminating time rule, but possibly dependent processing (i.e., possible race models would have to lie within this area); accordingly, the dashed line to the left shows the Miller and the right the Grice bound (upper and lower performance limits, respectively).

Experiment 1 yielded a significant difference between response times (RT) to unimodal V and O stimuli, indicating only little overlap between the unimodal RT distributions; V stimuli were perceived 362 ms faster than O stimuli [*t*_(6)_ = −4.72, *p* < 0.01; Figure 3A]. By contrast, we could observe a markedly reduced difference between the SOA-corrected unimodal RTs in Experiment 2 of only 74 ms [*t*_(6)_ = −2.48, *p* < 0.05; Figure 3B], indicating a strong overlap of unimodal RT distributions.

These performance differences were clearly reflected in the cumulative RT distributions: While the bimodal distribution mostly followed the visual distribution in Experiment 1, it was shifted toward faster responses throughout its whole range in Experiment 2 (Figures 3C,D).

We next tested whether the bimodal RT speedup could be explained by statistical facilitation in a separate-activation model with unlimited capacity (race model). The theoretical upper performance limit was given by the *Miller bound*: If observed responses were *faster* than this boundary at any time, all race models could be ruled out at once, and the system would be assumed to be super-capacity at this time (Townsend and Nozawa, 1995; Townsend and Wenger, 2004). Additionally, we compared our data to a lower performance bound proposed by Grice et al. (1984b), referred to as *Grice bound*. It assumes that responses in the bimodal situation should be at least as fast as responses to the fastest unimodal constituents. If responses were *slower* than this boundary, the system would be assumed to be limited-capacity (Townsend and Wenger, 2004) at this time. Both super-capacity and limited-capacity processing violate the assumption of context-invariance, invalidating an essential requirement of race models (Colonius, 1990). We found that the observed bimodal RTs did not exceed the *Miller* or the *Grice bounds* significantly in both experiments, indicating parallel processing of the visual and olfactory channels (Figures 3E,F, and Table 1). Our data could thus be attributed for by a race model.

**Table 1:** **Each participant contributed one quantile value, following the procedure from Ulrich et al. (2007)**.

**Experiment**	**Quantile (%)**	**VO–*****Miller bound***			**VO–*****Grice bound***			**VO–UCIP**		
		**Δ (ms)**	***t***	***p***	**Δ (ms)**	***t***	***p***	**Δ (ms)**	***t***	***p***
1	5	−20	−1.68	0.144	−20	−1.68	0.145	−20	−1.68	0.144
	15	−11	−1.15	0.295	−13	−1.52	0.180	−11	−1.18	0.281
	25	0	0.04	0.971	−13	−1.99	0.094	−2	−0.27	0.800
	35	8	0.82	0.443	−8	−1.43	0.204	5	0.58	0.583
	45	22	1.30	0.242	−4	−0.36	0.732	14	0.92	0.391
	55	45	2.30	0.061	2	0.11	0.915	33	1.85	0.114
	65	74	2.65	0.038	−6	−0.21	0.844	43	1.79	0.123
	75	111	4.26	0.005	−33	−1.05	0.333	50	2.62	**0.039**
	85	203	4.11	0.006	−118	−1.84	0.116	73	1.77	0.128
	95	337	4.41	0.004	−72	−0.91	0.400	74	0.94	0.384
2	5	13	0.77	0.472	11	0.65	0.543	13	0.77	0.473
	15	14	0.92	0.395	4	0.24	0.822	13	0.86	0.422
	25	29	2.01	0.091	9	0.52	0.622	26	1.71	0.138
	35	39	2.20	0.070	−13	−1.26	0.255	33	1.99	0.093
	45	46	3.98	0.007	−46	−3.05	0.022	31	3.49	**0.013**
	55	60	4.08	0.007	−69	−2.54	0.044	31	3.01	**0.024**
	65	77	5.08	0.002	−83	−2.92	0.027	29	2.92	**0.027**
	75	110	5.92	0.001	−112	−3.39	0.015	35	4.82	^*^0.003
	85	179	6.16	0.001	−120	−2.39	0.054	64	3.47	**0.013**
	95	293	4.89	0.003	−131	−2.09	0.082	54	1.37	0.218

*Negative t-values for the Miller and positive t-values for Grice bound comparisons indicate violations of the respective bounds. Significant model violations in bold; the asterisk marks a significant violation after Holm-Bonferroni correction for multiple testing*.

Colonius (1990) pointed out that the *Miller* and *Grice bounds* can only be reached under the implicit assumption of perfect negative and positive, respectively, dependence between channel processing speeds. To gain further insight into the underlying processing mechanisms, we compared our data to a model assuming uncorrelated processing between the visual and olfactory channels, the so-called *unlimited-capacity, independent, parallel* (UCIP) model. The bimodal RTs were slower than predicted by this model in the 75 % quantile in Experiment 1 and from the 45 % to the 85 % quantiles in Experiment 2 (all *p* < 0.05). However, only the deviation in the 75 % quantile in Experiment 2 survived Holm-Bonferroni correction for multiple testing (*p*_corr_ = 0.03). All comparisons are summarized in Table 1. Because the deviations of the observed data from the model predictions are shifted in direction of the *Grice bound*, i.e., toward perfect positive dependence, the results suggest a race model with positively correlated channel processing speed between the visual and olfactory channels (see Grice et al., 1984a).

**Figure 4:**
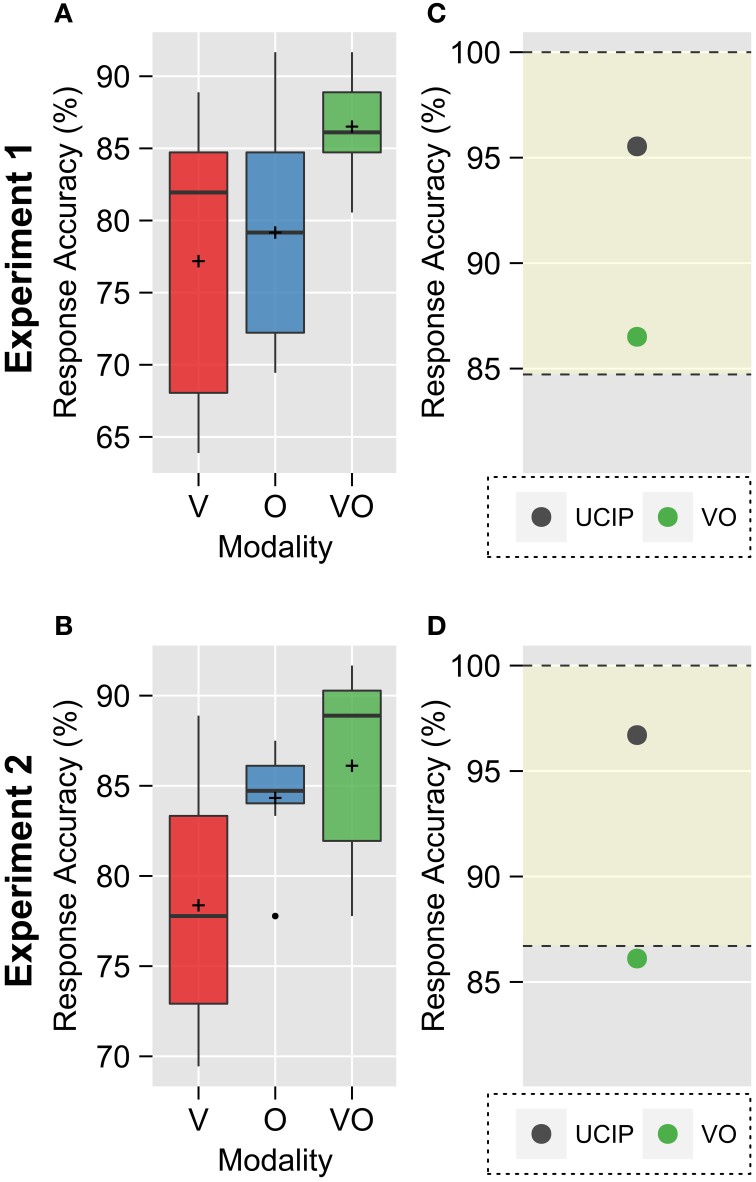
**Comparison of observed accuracies in the visual (V), olfactory (O), and bimodal (VO) trials**. **(A,B)** Mean VO accuracy is higher than either unimodal accuracy in both experiments, with mean V responses being the least accurate. **(C,D)** Comparison of the unlimited capacity, independent, parallel (UCIP) model prediction with the observed bimodal data. The highlighted area depicts the expected bimodal accuracy range under the assumption of separate-activation models with unlimited capacity, but possibly dependent processing. The cross in the boxplots depicts group means.

Next, the accuracy data (grand means shown in Figures 4A,B for Experiments 1 and 2, respectively) were compared to models of probability summation. We first adopted equivalents of the *Miller* and *Grice bounds* to derive upper and lower performance limits, respectively (Colonius, 2015). The upper bound was at 100 % accuracy in both experiments and therefore never violated; observed accuracies were significantly below this bound [Experiment 1: Δ = −13.5%-points, *t*_(6)_ = −8.95, *p* < 0.001; Experiment 2: Δ = −13.9%-points, *t*_(6)_ = −6.76, *p* < 0.001]. The lower bound was never significantly violated [Experiment 1: Δ = 1.8%-points, *t*_(6)_ = 0.73, *p* = 0.49; Experiment 2: Δ = −0.6%-points, *t*_(6)_ = −0.29, *p* = 0.78. Note that the lower bound was identical to the baseline used earlier to identify an RSE for accuracy. These results suggest that probability summation could in fact explain the observed bimodal accuracies.

The data were then compared to a model predicting stochastic independence, equivalently to the UCIP model employed for RTs (Stevenson et al., 2014). Bimodal accuracy was lower than predicted by the model in both Experiment 1 [Δ = −9.0%-points, *t*_(6)_ = −5.97, *p* < 0.001; Figure 4C] and Experiment 2 [Δ = −10.6%-points, *t*_(6)_ = −4.99, *p* < 0.01; Figure 4D]. In line with the RT data, the accuracy data indicate that the visual and olfactory channels are stochastically dependent.

## 3. Discussion

The present study found a bimodal response facilitation for perceptually, but not for physically aligned bimodal visual-olfactory stimuli. The facilitation could be accounted for by race models assuming probability summation across positively dependent processing channels. Thus, the results yielded no proof of coactivation.

The observation of a significant bimodal visual-olfactory response speedup indicated by an RSE for perceptually, but not for physically aligned unimodal constituents suggests that temporal proximity subserves visual-olfactory response facilitation. Increasing temporal parity amplifies multisensory interactions in other sensory modalities, e.g., for visual-tactile (Forster et al., 2002) or visual-auditory (Lovelace et al., 2003) stimuli albeit the temporal binding window, i.e., the range of inter-stimulus intervals over which multisensory stimuli are integrated, is not universal. While multisensory binding windows as large as several hundred milliseconds exist for example in audio-visual speech perception (see e.g., van Wassenhove et al., 2007), the effects of stimulus timing on visual-olfactory perception are unknown. To our knowledge, this is the first study demonstrating that perceptual, rather than physical, simultaneity is vital to elicit an RSE for bimodal visual-olfactory objects.

However, the response speedup could be the result of statistical facilitation alone and is not necessarily proof of neural integration processes. Therefore, we examined whether the present data could be explained by race models, or if we could find evidence for coactivation.

Response time distributions never significantly exceeded the *Miller bound*. We can therefore exclude coactivation as a possible explanation of the observed RSEs. Further we can exclude strictly limited-capacity processing over an extended period of time because the *Grice bound* was never violated (Townsend and Wenger, 2004). Taken together, the observed bimodal response times are consistent with separate-activation models with a first-terminating time rule and unlimited-capacity processing, i.e., race models (Miller, 1982; Grice et al., 1984a; Colonius, 1990).

Classically, it has been shown that violations of the *Miller bound* are more easily produced in go/no-go tasks due to the absence of “response competition” (Grice and Canham, 1990; Grice and Reed, 1992). Yet, race models can successfully be rejected in choice response time studies as well (see e.g., Miller, 1982; Hecht et al., 2008; Girard et al., 2011).

No change in response accuracy was observed in the bimodal conditions, compared to unimodal stimulation. This finding is in contrast to previous reports of improved accuracy for multisensory stimuli. A possible reason for this discrepancy might be that olfaction and vision do not integrate in the same way as other senses. However, it is also possible that we were not able to observe improved accuracy simply for statistical reasons due to the low number of trials (owing to the long inter-trial intervals, ITIs, necessary for olfactory stimuli) and high inter-subject variability.

Comparison of the observed bimodal response time distributions to a more restrictive race model assuming stochastic independence of channel processing speed (UCIP model) revealed significantly slower responses than predicted in both experiments, suggesting positively dependent channel processing speeds between the visual and olfactory channels (Grice et al., 1984a). Although only the deviation in one quantile in Experiment 2 was significant after correction for multiple testing, the additional finding of lower bimodal response accuracies than predicted further corroborates the assumption of a possibly positive stochastic dependence of visual and olfactory processing.

In contrast, the bimodal combination of odor and taste stimuli yielded faster responses than predicted by a UCIP model in a recent study (Veldhuizen et al., 2010). Notably, odor and taste perception are closely intertwined; evidence exists for direct and indirect anatomical connections between the primary gustatory and olfactory cortices (Rolls and Baylis, 1994; Shepherd, 2006) as well as for convergence areas responding to both smell and taste, for example in the orbitofrontal cortex (OFC) (O'Doherty et al., 2001; de Araujo et al., 2003; Small and Green, 2012), the anterior insula, and frontal and parietal opercula (Small et al., 1999; Cerf-Ducastel and Murphy, 2001; Poellinger et al., 2001). Perceptually, the combined odor-taste experience typically exceeds the sum of the two chemosensory modalities, being perceived as more *Gestalt*-like, intense and rewarding, and yields superadditive activation in the frontal operculum (Seubert et al., 2015). Although no monosynaptic connection between the primary visual and olfactory cortices has been found, the perirhinal cortex is a prime candidate as a processing hub between the visual and olfactory modalities due to its numerous reciprocal connections, particularly with the inferior temporal cortex. The inferior temporal cortex is involved in object perception (Grill-Spector and Weiner, 2014) and associations of sensory representations, and a subdivision, the rhinal cortex, has been proven critical for the association of flavor with visual food objects in monkeys (Parker and Gaffan, 1998).

### 3.1. Conclusion

The present data are consistent with models of parallel processing with unlimited-capacity and positive dependence between the visual and olfactory channels. Notably, these models do not refute the possibility of coactive processing. Although odor perception is highly ambiguous and susceptible to other sensory information (Herz and von Clef, 2001), the olfactory stimuli may in fact have contributed to the bimodal object identification by generating further perceptual evidence, allowing an internal decision criterion to be reached faster. This assumption is supported by the observation of positive channel dependence, indicating that the identification of the visual and olfactory constituents in bimodal trials co-occurs. The objects used in the present study carried a semantic meaning, which had to be decoded before mapping it to the appropriate response button. Semantic representations emerge only at later stages in the perception process (Olofsson, 2014). Further, no direct connections between the visual and olfactory cortices have been discovered yet, questioning the plausibility of early bimodal visual-olfactory interactions. Future studies will have to show whether the present findings are transferable to other stimulus objects, SOAs, and experimental tasks.

## 4. Materials and methods

### 4.1. Participants

Eight participants completed the study; one participant was excluded because his accuracy was far below chance level for the unimodal olfactory lemon stimulus in both experiments (mean accuracy was approx. 33 %); data of seven participants (4 female; age in years: 29.9±2.4SD, range: 26–32; all right-handed) are reported here. Participants were recruited from the German Institute of Human Nutrition and local universities; they gave written informed consent and received compensatory payment. They reported no neurological disorders or chronic diseases, in particular no smell impairment, and normal or corrected-to-normal vision. The study was conducted in accordance with the requirements of the revised Declaration of Helsinki and had been approved by the ethics committee of the German Society for Psychology (DPGs).

### 4.2. Stimuli

#### 4.2.1. Visual stimuli

Six images (three different images of bananas and lemons, respectively) with different complexities were selected from the Food-pics database (no. 276, 282, 341, 379, and 415; Blechert et al., 2014) or purchased online. Images displayed a food object centered on a white background. They were resized to 1024 × 1024 pixels and converted to grayscale. A Gaussian blur (order 0, σ = 3) was applied to remove sharp edges. The fast Fourier transform (FFT) of all images was calculated and the phase space was randomly scrambled. The inverse FFT of the image with the scrambled phase yielded blurry images of the food objects with superimposed cloud-like noise patterns. Noise-only images were also derived for every object using the same method, yielding 2 × 3 target and 2 × 3 noise-only stimuli in total. The spatial frequency of those noise patterns was similar to the spatial frequency of the original object. Images were presented on a TFT monitor with a resolution of 1680 × 1050 pixels. The refresh rate was set to 60 Hz. Participants viewed the images at an eye distance of approx. 60 cm, corresponding to an object size of approximately 12° of visual angle, embedded in visual noise of approximately27° of visual angle.

#### 4.2.2. Olfactory stimuli

Odorants were 10 mL aliquots of isoamyl acetate (banana; Sigma-Aldrich Chemie GmbH, Steinheim, Germany, CAS 123-92-2) and lemon oil (lemon; same vendor, CAS 8008-56-8) diluted with mineral oil (Acros Organics, Geel, Belgium, CAS 8042-47-5) to produce solutions of 0.1% v/v concentration. The solvent, pure mineral oil, served as neutral control. The odors were congruent to the visual objects banana and lemon; odor intensity was chosen to yield identifiable, yet weak stimuli based on a pilot study (*n* = 7). Odorants were presented birhinally using a custom-built 16-channel air-dilution olfactometer (Lundström et al., 2010). Teflon tubes with an inner diameter of 1/16′′ delivered the odorous air via custom-made anatomically shaped nose pieces into the participants' nostrils. A constant flow of clean air (approximately 0.5 L min^−1^) was present at all times to rinse the tubing system and the nose. Stimuli were delivered with a flow rate of approximately 3.0 L min^−1^, totaling to a flow of about 3.5 L min^−1^ during stimulation. Stimulus timing was measured using a photo-ionization detector (PID; 200B miniPID, Aurora Scientific Inc., Aurora/ON, Canada) and defined as the time point 254 ms after sending the trigger to the olfactometer. To ensure a constant odor concentration and to reduce depletion of head space in the odor jars in the course of the experiment, one of three identical odor jars was used in sequential order from trial to trial.

## 4.3. Procedure

Participants completed two experimental sessions on separate days. In the first session, a visual identification threshold assessment was conducted, followed by a choice response time (CRT) Experiment in which bimodal stimulus components were presented physically simultaneous. A second CRT experiment with perceptually aligned bimodal stimuli was conducted during the next session. The experiments were carried out in a sound-attenuated experimental booth. Participants were seated centered in front of the screen. Responses were collected using a button box (Serial Response Box, Psychology Software Tools, Sharpsburg/PA, USA) connected to a USB port of the stimulation computer via a serial-to-USB adapter. Timing accuracy was verified to be better than 2 ms. In-ear headphones delivered Brownian noise during the CRT experiments at a volume chosen such that the change in air flow at stimulation on- and offset was inaudible. The stimulation was controlled using PsychoPy 1.79.01 (Peirce, 2009) running on a personal computer.

### 4.3.1. Visual threshold estimation

We adjusted the strength of the noise so that objects could be perceived approximately on every second trial using a QUEST staircase procedure (Watson and Pelli, 1983). The Experiment started with a short practice block, in which all target and noise-only images were presented once. Then, images of objects + noise were presented interleaved with noise-only images (equal proportions) for 900 ms with a randomly varied ITI between 1.5 and 2.0 s during which a white screen was presented. Participants indicated by button press the detection of an object within the noise. The staircase adjusted the strength of the noise to yield a performance level of 50 % correct object detection when stimuli were present (false alarms on noise-only trials were very rare, ranging from 0 to approx. 3 %, with a grand mean of 1.3%.). Separate staircases were run for each of the six different object images. Overall, the threshold procedure entailed 240 trials, 20 repetitions of each of the six images and their respective noise-only images (2 × 6 images × 20 repetitions). Participants were allowed a short break; the procedure lasted about 12 m. Note that stimuli yielding 50 % accuracy in this detection task are expected to yield approximately 75 % performance in the 2-AFC task as used in the main experiment.

### 4.3.2. Bimodal CRT experiments

During the CRT experiments, participants were to identify the presented object (banana or lemon) as quickly as possible (while avoiding anticipatory responses) by pressing either of two buttons on the button box. Stimuli were either unimodal visual (V) objects presented at individual 50% identification threshold, unimodal olfactory (O) objects, or bimodal visual-olfactory (VO) objects. V stimuli were always paired with the neutral control odorant. O stimuli were paired with a randomly assigned noise-only image derived from a visual stimulus of the same object. OV stimuli consisted of the combined presentation of congruent V and O stimuli.

VO stimulus pairs were presented simultaneously in Experiment 1. In Experiment 2, bimodal stimulus timing was adjusted to achieve perceptual simultaneity by introducing an SOA equal to the difference of unimodal median RTs individually for each object and participant. The mean SOAs were 330±295msSD for banana, and 395±205msSD for lemon. Note that all SOAs were positive, i.e., delaying visual presentation, except for banana in one participant, where the odor had to be presented 182 ms prior to the visual stimulus to achieve perceptual simultaneity. To ensure context-invariance in Experiment 2, we also adjusted the timing of the unimodal stimulus presentations. Specifically, if the estimated SOA indicated a delayed presentation of the visual constituent in bimodal conditions, we also delayed the visual stimulation in the unimodal conditions for the same amount of time (meaning the fixation cross was visible for a longer duration before the stimulus appeared; note that this was also true for the unimodal olfactory stimulation, where the visual stimulus was noise-only). The stimulus timing is illustrated in Figure 1.

Each trial started with a fixation cross centered on the screen, which informed participants to prepare and to slowly inhale. At the same time, the air flow through the neutral jar was initiated to remove the tactile cue from the later stimulus presentation. After a random period of 1–2 s, a stimulus (O, V, or VO) was presented for 900 ms. After stimulation, the neutral control odorant was presented for 4.1 s to remove residual odor molecules. The ITI was randomly varied between 20 and 21 s.

The experiments started after a short practice block in which each stimulus combination was presented once. Each Experiment consisted of six blocks during which all stimulus combinations were presented twice and in pseudo-random order, totaling to 216 stimuli (6 blocks × 2 repetitions × (6 V+6 O+6 VO)), and lasted 95–120 min. Participants were allowed self-paced breaks in the middle of each block and between blocks.

RT measurement started with the onset of the image in V trials and the physical onset of the odorant as determined by PID measurements in O trials. In bimodal trials, RT measurement started with the physical onset of the stimuli (Experiment 1, physically simultaneous presentation), or with the onset of the earlier stimulus (Experiment 2, perceptually simultaneous presentation).

## 4.4. Data analysis

Only trials with positive and correct identification responses were analyzed. RT medians and standard deviations (SDs) of the aggregated data were calculated for each of the six conditions (O, V, VO for banana and lemon objects, respectively). All trials with a reaction time deviating more than two SDs from the median were discarded as outliers. In Experiment 1 and 2, 6.0% and 5.5% of the trials were removed, respectively.

A short summary of the analyses will be given in the next paragraph, followed by a detailed method description in the remaining section.

Faster responses to bimodal, compared to unimodal, stimuli indicate an RSE. Therefore, we first compared bimodal to unimodal RTs by calculating the difference between the bimodal and the faster of the two unimodal RTs (visual or olfactory). Because this global RSE is relatively insensitive to effects that are not present across the whole response time range, we next estimated cumulative distribution functions (CDFs) from the RTs. Analyses based on these CDFs can take into account the whole RT distribution. We evaluated the CDFs at 10 quantiles. Since an observed response speedup can be caused by statistical facilitation alone, in a next step we calculated theoretical model boundaries based on the unimodal CDFs under the assumption of parallel processing of the visual and olfactory channels (race model), that is the data range that could be explained by statistical facilitation. Any observation exceeding these limits would support the hypothesis of true integrative processing. To examine whether the channels operated in a stochastically independent manner, we additionally compared our data to a very specific race model assuming stochastic independence of the channel processing speeds (UCIP model). A very similar approach was chosen in the analysis of the accuracy data, although it was naturally based on mean accuracies and not single-trial responses, i.e., no equivalent of a CDF could be estimated.

### 4.4.1. Response times

The RT distributions were heavily positively skewed; we therefore used the median as measure of central tendency. This measure is not without criticism (cf. Miller, 1988), but alternatives like the commonly applied log-transformations are not universally applicable approaches either (Feng et al., 2014).

Response times to unimodal V and O stimuli and their bimodal VO combination are defined as non-negative random vectors RT_*V*_, RT_*O*_ and RT_*VO*_. Their respective expected values shall be labeled *E*(RT_*V*_), *E*(RT_*O*_) and *E*(*RT*_*VO*_), and their distribution functions as *F*(RT_*V*_), *F*(RT_*O*_), and *F*(RT_*VO*_). An RSE can be observed if

(1) $$E({\text{RT}}_{VO})<\min[E({\text{RT}}_{V}),E({\text{RT}}_{O})],$$

i.e., if mean RTs for bimodal VO stimuli are faster than for either unimodal component.

We calculated the difference between the medians of the fastest unimodal and the bimodal RTs, i.e., min[*E*(RT_*V*_), *E*(RT_*O*_)]−*E*(RT_*VO*_). Positive values indicate a bimodal speedup, i.e., a facilitation in processing of bimodal as compared to unimodal stimuli. Note that RSEs were calculated separately for each object (banana and lemon) before collapsing and submission to one-sample *t*-tests against zero to identify bimodal facilitation.

To quantify the effect of in perceptually aligning the unimodal constituents of bimodal trials in Experiment 2, we compared the median RTs of the unimodal V and O conditions (collapsed across objects) using paired *t*-tests for each experiment.

Next, we tested whether the RT distributions fit probability summation models. In bimodal trials, only the marginal distribution *F*(RT_*VO*_), but not the distributions of the unimodal constituents *F*(RT_*V*_) and *F*(RT_*O*_) can be observed.

Probability summation models critically rely on the assumption of *context-invariance* (Colonius, 1990), which states that the processing speed of a channel is identical in unimodal and bimodal stimulations, that is additional work load on one channel does not influence processing speed in another channel, suggesting *unlimited capacity*.

The unlimited capacity, independent, parallel (UCIP) model makes the additional assumption that the processing speeds of individual channels are uncorrelated and hence *stochastically independent* (Raab, 1962; Meijers and Eijkman, 1977). According to a UCIP model, the cumulative distribution function for the bimodal stimulation is:

(2) $$F({\text{RT}}_{VO})(t)=F({\text{RT}}_{V})(t)+F({\text{RT}}_{O})(t)-F({\text{RT}}_{V})(t)\times F({\text{RT}}_{O})(t).$$

The last term is always equal to or greater than zero, i.e., *F*(RT_*V*_)(*t*) × *F*(RT_*O*_)(*t*) ≥ 0.

Miller (1982) discarded the assumption of stochastic independence and instead assumed a maximally negative dependence between the channel processing speeds (Colonius, 1990). This allowed him derive an upper bound for the maximum achievable performance gain under *any* parallel processing model called *Miller bound* or *race model inequality*(RMI), commonly expressed as:

(3) $$F({\text{RT}}_{VO})(t)\leq F({\text{RT}}_{V})(t)+F({\text{RT}}_{O})(t)$$

All parallel processing models have to satisfy inequality (3). If the inequality is violated, the assumption of parallel processing must be dropped, i.e., all race models are ruled out immediately, and the results can only be accounted for by what Miller called *coactivation models* (Miller, 1982)^1^. Similarly, a lower performance bound was defined by Grice et al. (1984b), implying perfect positive dependence (Colonius, 1990) between the channels' processing speeds:

(4) $$F({\text{RT}}_{VO})(t)\geq \max[F({\text{RT}}_{V})(t),F({\text{RT}}_{O})(t)]$$

That is, performance in the bimodal conditions should be equal to or faster than in the fastest unimodal condition.

In the case of asynchronous stimulation, i.e., by delaying the presentation of the visual stimulus by the time τ, Equations (1), (2), respectively, become (Miller, 1986):

(5) $$E({\text{RT}}_{VO(\tau )})<\min[(E({\text{RT}}_{V}+\tau ),E({\text{RT}}_{O})],\text{ and}$$

(6) $$\begin{array}{l} F({\text{RT}}_{VO(\tau )})(t)=F({\text{RT}}_{V})(t-\tau )+F({\text{RT}}_{O})(t)\\ -\;F({\text{RT}}_{V})(t-\tau )\times F({\text{RT}}_{O})(t). \end{array}$$

Note that the visual RT distribution *F*(RT_*V*_)(*t*−τ) is shifted to the right, which is the correct adjustment for the SOA. The adjusted *Miller* and *Grice bounds* from Equations (3), (4) can then be expressed as:

(7) $$F({\text{RT}}_{VO(\tau )})(t)\leq F({\text{RT}}_{V})(t-\tau )+F({\text{RT}}_{O})(t),\text{ and}$$

(8) $$F({\text{RT}}_{VO(\tau )})(t)\geq \max[F({\text{RT}}_{V})(t-\tau ),F({\text{RT}}_{O})(t)].$$

We estimated empirical cumulative distribution functions (CDFs) of the RTs using a Python implementation of the algorithm suggested by Ulrich et al. (2007). The CDFs predicted by the UCIP model denoted in Equation (6), as well as the theoretical race model boundaries from Equations (7), (8) were calculated based on the unimodal CDFs, resulting in six CDFs per participant (unimodal O and V, bimodal VO, UCIP model, upper and lower bound). All CDFs were then evaluated at ten evenly spaced quantile points (0.05, 0.15, …, 0.95), which were subsequently collapsed across both objects. The resulting values were submitted to separate paired *t*-tests for every quantile to test for deviations from the model predictions.

### 4.4.2. Accuracy

Similar to Equation (1), an RSE in accuracy can be observed if

(9) $$E({\text{ACC}}_{VO})>\max[(E({\text{ACC}}_{V}),E({\text{ACC}}_{O})],$$

i.e., if mean accuracy for bimodal VO stimuli is higher than for the most accurate of the unimodal components.

We calculated the difference between the means of the most accurate unimodal and the bimodal responses, i.e., *E*(ACC_*VO*_)−max[(*E*(ACC_*V*_), *E*(ACC_*O*_)]. Positive values indicate a bimodal accuracy enhancement. Note that RSEs were calculated separately for each object before collapsing to one-sample *t*-tests against zero.

Following the assumption of the UCIP model, Equation (2) can be applied to accuracy data and becomes (Stevenson et al., 2014):

(10) $$p({\text{ACC}}_{VO})=p({\text{ACC}}_{V})+p({\text{ACC}}_{O})-p({\text{RT}}_{V})\times p({\text{ACC}}_{O}).$$

Equivalents of the *Miller* and *Grice bound*s for bimodal accuracy were proposed by Colonius (2015). Formulas (3) and (4), respectively, then become

(11) $$p({\text{ACC}}_{VO})\leq p({\text{ACC}}_{V})+p({\text{ACC}}_{O}),\text{ and}$$

(12) $$p({\text{ACC}}_{VO})\geq \max[p({\text{ACC}}_{V}),p({\text{ACC}}_{O})].$$

The model predictions and boundaries were calculated for each Experiment and object separately. The results were then collapsed across objects. The resulting values were then submitted to paired *t*-tests to test for deviations from the model predictions.

## Author contributions

RH, NB, and KO designed the experiments; RH acquired the data and performed the analyses with input from KO and NB. RH and KO wrote the paper.

### Conflict of interest statement

The authors declare that the research was conducted in the absence of any commercial or financial relationships that could be construed as a potential conflict of interest.

## Footnotes

^1^However, it should be noted that the reverse is not true: Showing that the observations can be described using a parallel processing model does not necessarily exclude coactivation models.
